# Effect of mitomycin C on the secretion of granulocyte macrophages colonies stimulating factor and interleukin-5 in eosinophilic nasal polyps stromal culture

**DOI:** 10.1016/S1808-8694(15)31199-X

**Published:** 2015-10-20

**Authors:** Paulo Fernando Tormin Borges Crosara, Anilton César Vasconcelos, Roberto Eustáquio Santos Guimarães, Helena Maria Gonçalves Becker, Celso Gonçalves Becker, Sandra Letícia Reis Crosara, Evaldo Nascimento

**Affiliations:** 1 Substitute Professor.; 2 Joint Professor, Department of General Pathology, Federal University of Minas Gerais.; 3 Full Professor, Department of Ophthalmology, Otorhinolaryngology and Speech and Hearing Therapy, Federal University of Minas Gerais.; 4 Ph.D., Department of Ophthalmology, Otorhinolaryngology and Speech and Hearing Therapy, Federal University of Minas Gerais.; 5 Ph.D., Department of Ophthalmology, Otorhinolaryngology and Speech and Hearing Therapy, Federal University of Minas Gerais.; 6 Resident, Núcleo de Otorrino-BH.; 7 Joint Professor, Department of Parasitological Sciences, Federal University of Minas Gerais. CAPES. Study conducted at the Department of Ophthalmology, Otorhinolaryngology and Speech and Hearing Therapy, Federal University of Minas Gerais. Immunology Laboratory: Professor Evaldo Nascimento.

**Keywords:** polyposis, nasal, eosinophil, GM-CSF, IL-5, mytomicin C

## Abstract

The research involving tissue factors, such as granulocyte macrophage colonies stimulating factor (GM-CSF) and interleukin 5 (IL-5), leads to the mechanisms involved in the maintenance of eosinophilia, which is essential for the pathogenesis on eosinophilic nasal polyps. Mitomycin C has been successfully used in otolaryngology. **Aim**: The objective of this study was to evaluate the effect of mitomycin C in secretion of GM-CSF and IL-5 on eosinophilic nasal polyps. **Study design:** case-control. **Material and Method**: This is a comparative and auto-matched experimental study, performed with fragments of polyps which had been obtained from biopsy of patients with eosinophilic nasosinusal polyposis. The fragments of the experimental group were treated with mitomycin C (400 microg/ml) for 5 minutes and then washed in RPMI substrate. At time zero, 12 and 24 hours, the surface material was taken to determination of its GM-CSF levels in 22 patients and of IL-5 levels in 19 patients, by ELISA method. **Results:** Reduction in GM-CSF expression on the experimental group at time 24 h (p<= 0.05). The treated group presented significant GM-CSF expression between zero time and 12 h time (p=0.013) showing the culture viability such as in the non-treated group. Tendency to decreasing IL-5 levels on the treated groups at 24 hours. **Conclusion**: This study showed that mitomycin C was efficient in inhibiting GM-CSF synthesis with reduction of IL-5 secretion, but this fact needs complementary studies.

## INTRODUCTION

Nasosinusal polyposis - NSP results from a chronic inflammatory process of the nasosinusal mucosa, with different stages of nasosinusal cavity impairment. Eosinophilic NSP represents 80 to 90% of NSP, associated with nasal and/or bronchial hyperreactivity and responds well to corticotherapy. It involves patients with different etiological factors and clinical evolution relative to upper and lower airways. Eosinophilic NSP is found in Nonallergic rhinitis with eosinophilia syndrome (NARES), in Fernand-Widal syndrome, atopia, allergic fungal sinusitis and Churg Strauss syndrome. NSP affects adult subjects with prevalence of 1 to 4% of the population and it is frequently increase in view of the concomitance with non-allergic bronchial asthma, reaching peaks at about 40 years of age.^1^, ^2^

Treatments of choice for this affection are: use of corticoids, that act in the reduction of the inflammatory process determined by the eosinophils, and surgical treatment. Surgical indication is reserved for cases that demonstrate a significant obstruction of nasal cavities or those associated with recurrent infections of nasosinusal mucosa. Despite the relevant advances in basic research, little has progressed in therapeutic terms. The rate of recurrence is high, even after a surgical procedure.

The study of GM-CSF and IL-5 is justified by the fact that they are extremely important cytokines for differentiation, migration, capture, activation and survival of eosinophils. They are produced by eosinophils in high amounts in the inflammatory sites, resulting in a self-sustainable cycle of stimulation. Such cytokines, associated with eosinophils, acquire a key role in the pathophysiology of eosinophilic NSP.^2^, ^3^, ^4^, ^5^, ^6^

The use of MMC is extremely important because it is an antineoblastic and antibiotic drug that acts as an alkylating agent, inhibiting DNA, protein synthesis and fibroblasts in cell cultures. MMC has been experimentally used in the prevention of stenosis in maxillary antrostomies, glaucoma surgery and pterygiums, in myringotomy and in laryngeal stenosis.^7^, ^8^, ^9^, ^10^

The purpose of the present study was to assess the action of Mitomycin C over the synthesis of the granulocyte macrophage colony stimulating factor (GM-CSF) and interleukin 5 (IL-5).

## MATERIAL AND METHOD

The studied population was formed by patients with extensive NSP, aged between 16 and 79 years, referred by SUS (Universal Healthcare System in Brazil), to perform surgical treatment at Hospital Sao Geraldo, a unit of Hospital das Clínicas, Federal University of Minas Gerais - HCUFMG.

We selected 22 patients with eosinophilic NSP, with eosinophil percentage equal or greater than 40% ^11^, in the period between January and June 2003. Patients with non-eosinophilic polyposis such as cystic fibrosis, Kartagener syndrome, antro-choanal polyp or eosinophilic NSP with active infection were excluded from the study.

Selected patients were submitted to complete ENT examination, special attention to the nasal segment. Nasal fossa was explored via anterior rhinoscopy and fibronasolaryngoscopy.

We carried out an experimental comparative self-matched study with 22 samples of eosinophil polyps. The studied group comprised culture by fragment of polyps treated with MMS and assessed at zero, 12 and 24 hours for each patient. The control group comprised culture of fragments of polyps not treated with MMC and assessed at the same hours.

We carried out biopsy of nasal polyps both for characterization of eosinophilia and cultures. The fragments obtained for eosinophilic assessment were immediately fixed with formol at 10% and referred to histopathological exam with hematoxylin-eosin (HE). Findings of at least 40% of eosinophils in inflammatory cells and/or presence of at least four eosinophils per four microscopic fields of magnification were considered eosinophilia ^11^.

Biopsies of nasal polyps obtained for culture were made in the ambulatory of Otorhinolaryngology, HC-UFMG. Removed fragments were immediately placed in a culture at room temperature. Fragments were sectioned in three portions of 3mm^3^ and grew in a plate with 6 orifices containing 0.5ml RPMI medium. Among the six compartments, three formed the experimental group and three the control group. In the experimental group we applied MMC for 5 minutes, at dosage of 400 µg/ml.^9^ After the application of the drug, cultures were rinsed in RPMI medium. To the control group, not treated with MMC, we made the same manipulation only with RPMI medium. Fragments and culture media were placed in the first compartments, control and experiment were immediately frozen for later use with the method Enzyme Linked Immunosorbent Assay (ELISA). The other two pairs of samples, both containing control and experiment, were incubated for 12 and 24 hours, respectively, in hot oven at 37° C and 0.5% CO_2_ up to the moment of collection and freezing.

The used medium of culture was RPMI 1640, commercialized by Gibco UK, containing 5% human serum AB, 2 m M mercaptoethanol, 1 mM L-glutamin, 2mM sodium piruvate, 10m gr/ml streptomycin, 100U/ml penicillin and 5mg/ml fungisone.^12^

At zero, 12 and 24 hours, cultures were preserved at 80° C temperature up to the moment of the analysis. After defrosting at room temperature, cultures were centrifuged at 3° C during 10 minutes at 15,000 rpm.^12^ Supernatants were submitted to ELISA method to determine the levels of GM-CSF and IL5. Kits R&D Systems: Duoset^®^ Human GM-CSF / Catalog: DY215; Duoset^®^ Human IL-5 Catalog: DY205.^12^ Out of biopsied patients, 22 were submitted to analysis for quantification of GM-CSF and 19 for IL-5.

The method used for statistical analysis was the t paired test to compare the groups in each one of the times, according to Snedecor and Cochran (1977).^13^ We considered the level of significance of 5% and we used the statistical package Minitab, version 13, for statistical data analysis.

The study was previously approved by the Research Ethics Committee, UFMG.

## RESULTS

Out of 22 studied experiments to determine the levels of GM-CSF, in three of them we did not reach enough size for the culture at hour 24, and in 19 of them experiments were assessed at this time.

**Figure 1:**
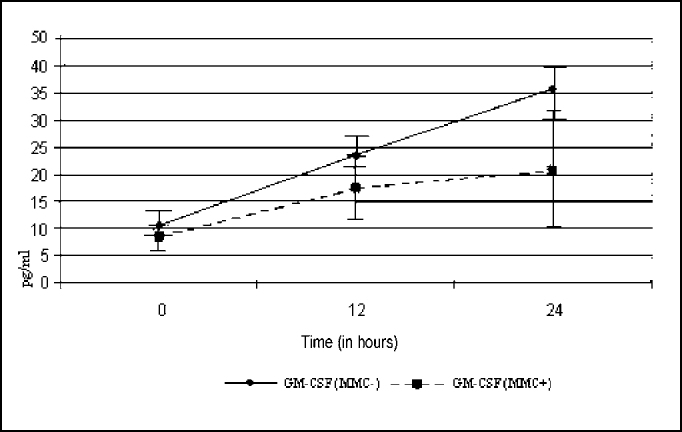
Curves of GM-CSF expression, with and without application of MMC.

Figure 1 demonstrates the expression of GM-CSF in cultures. We could observe that at zero hour, cultures, with or without MMC, did not present statistical difference between the two groups (p= 0.362). At hour 12, the behavior of the curve revealed that the group that received MMC presented lower levels of GM-CSF in relation to the control group, but there was no statistically significant difference (p= 0.570). At 24 hours, the curve showed statistically significant difference between the two groups (p £ 0.05).

Assessing separately the expression of GM-CSF in the two experiments, we observed that in the group without MMC, between zero and 12 hours, there was no statistically significant difference (p= 0.087). Between zero and 24 hours, there was statistically significant difference (p= 0.048). There was no statistically significant difference between hour 12 and hour 24 (p= 0.409), even though at hour 24 we had reached the same levels of GM-CSF.

Similarly, analyzing the group with MMC application, we observed similar behavior, with statistically significant differences for zero and 12 hours (p= 0.013) and borderline between zero and 24 hours (p= 0.068), and we did not observe statistically significant difference in the comparison between 12 and 24 hours (p= 0.606), even though levels of GM-CSF had been higher than 24 hours.

**Figure 2:**
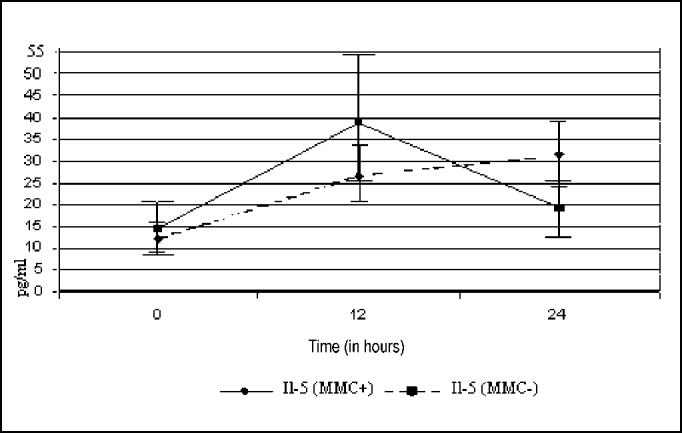
Curves of expression of IL-5, with and without application of MMC.

Figure 2 shows the expression of IL-5 in the studied cultures. Out of 19 experiments, two did not reach enough size for culture of 24 hours, and only 17 experiments were assessed at this time.

When compared at zero hour, we observed that there was no statistically significant difference between the groups (p= 0.362). The curve shows greater expression of IL-5 in the group treated with MMC, but this difference was not significant in 12 hours (p= 0.281), with clear drop in levels of IL-5 as of this time. Conversely, control cultures that were not treated with MMC had growing expression of IL-5, and we did not observe any significant difference between the two groups at 24 hours (p= 0.105).

Upon analyzing the two groups separately, we observed that in MMC cultures, there was statistically significant difference in expression of IL-5 between zero and 12 hours (p= 0.046), and between zero and 24 hours (p= 0.027). Between 12 and 24 hours, there was no statistically significant difference (p= 0.671). In the cultures treated with MMC, we did not find any significant difference between zero and 12 hours (p= 0114), zero and 24 hours (p= 0.635), as well as between 12 and 24 hours (p= 0.112).

## DISCUSSION

The study of eosinophil physiology has demonstrated that these cells perform an essential role in the allergic inflammatory and anti-parasitic response, presenting the capacity to interact with other cells through specific receptors. They are extremely versatile and produce a wide range of substances capable of causing tissue lesion and others that may expand and perpetuate the inflammatory response.^14^

Eosinophils have serum half-life of approximately 18 hours, but they can remain for weeks in the tissues, depending on environmental stimuli ^15^. Small eosinophilic grains are responsible for the accumulation of toxic substances, such as major basic proteins (MBP), eosinophilic cationic proteins (ECP) and eosinophilic peroxidase (EPO). They are involved in the cytotoxicity against parasites and tissues, such as we find in bronchial asthma, urticaria and eosinophilic NSP.^16^

Cara, Negrão-Corrêa and Teixeira (2000)^14^ made an excellent review about the physiology of eosinophils. According to the authors, eosinophils express receptors to many different molecules, such as: immunoglobulins (IgG, IgE, IgA), complement (C1q, C3b/C4b, iC3b, C5a), kemokines (eotaxin, eotaxin-2, RANTES, MCP-3, MCP-4), cytokines (IL-3, IL-5 and GM-CSF), lipid mediators (PAF, LTB_4_ ) and finally steroids (estrogen and glucocorticoids). Through these receptors, eosinophils may interact with the environment. In addition to these receptors, there are also clustering determining antigens (ADA), among which we can include cell adhesion molecules CD11/CD18, VLA-4,(±421), ±427 and CD62L (L-selectin).

In the last decade, eosinophils have been related to eosinophilic inflammatory diseases and there was no significant difference between pathologic processes of eosinophilia in NSP, intrinsic or extrinsic asthma. In many different studies, eosinophils are considered the main cells involved in the genesis of these diseases.^5^, ^6^ Thus, in these studies, we considered to be value any type of eosinophilic NSP, and there was no distinction among the studied cases. Kramer et al. (2000)^17^ suggested that the mechanisms involved in the genesis of eosinophilic polyposis, of allergic origin or not, are similar.

Clutterbuck, Hirst and Sanderson (1989)^18^, using recombining IL-5, IL-3 and GM-CSF in human bone marrow cultures observed that IL5 stimulates specifically the differentiation of eosinophils, without influencing other lineages such as eosinophils/basophils or eosinophils/erythrocytes. IL-3 and GM-CSF in high concentration, both stimulate the eosinophil colony such as netrophils and mixed colonies of eosinophils and granulocyte macrophage. Working together, IL-5, IL-3 and GM-CSF present an additive and non-synergetic effect, even in low concentrations of IL3 and GM-CSF.

According to Sanderson (1992)^19^, IL-5 is important and specific in the differentiation of eosinophils. In the same manner, the author has genetically manipulated mice, managing to have a line with increase in production of IL-5 and findings of hyper-eosinophilia. The opposite experience was developed by Foster et al. (1996)^20^ and Kopf et al. (1996)^21^, in which the line of mice with low level of IL5 resulted in hypo-eosinophilia, even after stimulation with allergens. It is known that both isolated or in synergism with IL-5, eotaxin is capable of releasing eosinophils from the bone marrow to the blood stream.^22^

Lacy and Moqbel (1997)^23^ concluded that the importance of the synthesis of GM-CSF for eosinophils resides in autocrine and paracrine actions, capable of activating and prolonging the survival of their own in vivo eosinophils.

MMC is an alkylating antiblastic agent discovered by Hata et al. (1956)^24^ and it acts in the inhibition of DNA synthesis.^25^ It was capable of inducing apoptosis in gastric tumor cells.^26^ Kim et al. (1999)^27^ observed that MMC (0.4 mg/ml for five minutes) induced in vitro apoptosis of human fibroblasts in Tenon capsule.

ELISA test has shown high sensitivity and specificity in many different studies involving eosinophilic NSP with IL-5 and GM-CSF investigation.^2^, ^3^, ^4^, ^17^

The results obtained in this assay showed that the levels of GM-CSF were significantly lower in the cultures treated with MMC within 24 hours, (p£ 0.05). Probably, owing to size of the involved sample (n=22), it was not possible to detect significant difference within 12 hours, but owing to higher accumulation of cytokines within 24 hours, we found statistically significant difference between zero and 12 hours (p= 0.013) and borderline between zero and 24 (p= 0.068) for cultures treated with MMC.

In the group not treated with MMC, there was the same pattern of accumulation, with statistical difference between zero and 24 hours (p= 0.048). It was not possible to detect a statistically significant difference between 12 and 24 hours in both groups (p= 0.606 - with MMC) and (p= 0.409 - without MMC), probably owing to size of sample involved (n= 19), which was not expressive enough for statistical detection in these times. These results are in agreement with the literature, which demonstrates reduction of the expression of GM-CSF when using corticosteroids in eosinophilic NSP.^28^, ^29^, ^30^

The results of the action of MMC in IL-5 synthesis did not detect any significant difference between the two groups, with or without MMC treatment in times zero, 12 and 24 hours. The p values were: p= 0.362, p= 0.281 and p= 0.105, respectively.

Upon observing the curve of IL-5 secretion only in the group treated with MMC, we detected higher initial production of cytokine at 12 hours and sudden drop at 24 hours. In this same groups, owing to large variation of results, with large standard deviation, we did not observe difference between times zero and 12 hours (p= 0.114), zero and 24 hours (p= 0.635) and 12 and 24 hours (p= 0.112). In Figure 2, we observed that the levels of secretion IL5 quickly drop between 12 and 24 hours, which suggests an action of the drug in this group.

As to IL-5, we did not observe the action of MMC as expected in the objectives, even with tendency to reduction of levels of cytokines (Figure 2). However, given that there was no previous control of size of samples, we could not conclude that MMC has had no action over IL-5 secretion, requiring further study.

The non-treated group with MMC proved to be more homogenous, with similar results to those found in GM-CSF study. There were statistically significant differences between zero and 12 hours (p= 0.046) and between zero and 24 hours (p= 0.027). We did not observe statistically significant difference between 12 and 24 hours (p= 0.671), probably owing to lack of nutrients in the culture. We observed that the cultures remained viable, with growing production of IL-5.

## CONCLUSION

The data in the present study show that mitomycin C was capable of significantly inhibiting the synthesis of GM-CSF. Data obtained in expression of IL-5 indicate a strong tendency to the reduction of this cytokine induced by mitomycin C.
